# Pharmacokinetics and pharmacodynamics of a single dose Nilotinib in individuals with Parkinson's disease

**DOI:** 10.1002/prp2.470

**Published:** 2019-03-12

**Authors:** Fernando L. Pagan, Michaeline L. Hebron, Barbara Wilmarth, Yasar Torres‐Yaghi, Abigail Lawler, Elizabeth E. Mundel, Nadia Yusuf, Nathan J. Starr, Joy Arellano, Helen H. Howard, Margo Peyton, Sara Matar, Xiaoguang Liu, Alan J. Fowler, Sorell L. Schwartz, Jaeil Ahn, Charbel Moussa

**Affiliations:** ^1^ Translational Neurotherapeutics Program Laboratory for Dementia and Parkinsonism Department of Neurology Georgetown University Medical Center Washington District of Columbia; ^2^ Movement Disorders Clinic Department of Neurology MedStar Georgetown University Hospital Washington District of Columbia; ^3^ Department of Biostatistics, Bioinformatics and Biomathematics Georgetown University Medical Center Washington District of Columbia; ^4^ Department of Pharmacology Georgetown University Medical Center Washington District of Columbia

**Keywords:** alpha‐synuclein, dopamine, Nilotinib, Parkinson, TREM2

## Abstract

Nilotinib is a broad‐based tyrosine kinase inhibitor with the highest affinity to inhibit Abelson (c‐Abl) and discoidin domain receptors (DDR1/2). Preclinical evidence indicates that Nilotinib reduces the level of brain alpha‐synuclein and attenuates inflammation in models of Parkinson's disease (PD). We previously showed that Nilotinib penetrates the blood‐brain barrier (BBB) and potentially improves clinical outcomes in individuals with PD and dementia with Lewy bodies (DLB). We performed a physiologically based population pharmacokinetic/pharmacodynamic (popPK/PD) study to determine the effects of Nilotinib in a cohort of 75 PD participants. Participants were randomized (1:1:1:1:1) into five groups (n = 15) and received open‐label random single dose (RSD) 150:200:300:400 mg Nilotinib vs placebo. Plasma and cerebrospinal fluid (CSF) were collected at 1, 2, 3, and 4 hours after Nilotinib administration. The results show that Nilotinib enters the brain in a dose‐independent manner and 200 mg Nilotinib increases the level of 3,4‐Dihydroxyphenylacetic acid (DOPAC) and homovanillic acid (HVA), suggesting alteration to dopamine metabolism. Nilotinib significantly reduces plasma total alpha‐synuclein and appears to reduce CSF oligomeric: total alpha‐synuclein ratio. Furthermore, Nilotinib significantly increases the CSF level of triggering receptors on myeloid cells (TREM)‐2, suggesting an anti‐inflammatory effect. Taken together, 200 mg Nilotinib appears to be an optimal single dose that concurrently reduces inflammation and engages surrogate disease biomarkers, including dopamine metabolism and alpha‐synuclein.

## 1 INTRODUCTION

Nilotinib (Tasigna^®^, AMN107, Novartis, Switzerland) is known as a break point cluster (BCR)‐Abl (Abelson) tyrosine kinase inhibitor approved by the U.S. FDA for adults with chronic myeloid leukemia (CML) at oral doses of 600‐800  mg daily.^1^, ^2^, ^3^ Nilotinib is also a potent inhibitor of discoidin domain receptors (DDR) 1 and 2.^4^, ^5^ We previously demonstrated that a low dose of Nilotinib (1‐10  mg/kg daily) penetrates the blood‐brain barrier (BBB), reduces inflammation and degrades misfolded alpha*‐*synuclein in several animal models of neurodegenerative diseases.^6^, ^7^, ^8^, ^9^ Nilotinib also increases dopamine (DA) levels and improves motor and cognitive outcomes in Parkinson's disease (PD) and Alzheimer's disease (AD) models.^6^, ^9^, ^10^, ^11^, ^12^, ^13^, ^14^, ^15^ In a small, 12‐patient pilot study, Nilotinib appeared to be potentially effective in treating the motor and nonmotor symptoms in patients with PD and dementia with Lewy bodies (DLB) and seemed to affect surrogate disease biomarkers, including DA metabolism and alpha‐synuclein.^16^

PD is the second most common neurodegenerative disorder that causes motor and nonmotor symptoms. PD is characterized by loss of DA‐producing neurons in the substantia nigra (SN) *pars compacta* and formation of intracellular inclusions known as Lewy bodies (LBs) that primarily contain aggregated alpha‐synuclein. Cerebrospinal fluid (CSF) levels of alpha‐synuclein oligomers longitudinally increase in PD compared to aged‐matched controls.^17^, ^18^, ^19^ Additionally, the ratio of oligomeric to total alpha‐synuclein also increases in the CSF of PD patients when compared to control and this increased ratio has been associated with motor decline.^20^, ^21^ Homovanillic acid (HVA) and 3,4‐Dihydroxyphenylacetic Acid (DOPAC) are two primary metabolites of DA and can be used as a CSF marker of DA metabolism. Decreased CSF levels of DOPAC have been shown to be an early marker for PD,^22^ and similarly HVA has been shown to be decreased in the CSF of PD patients compared to controls.^23^ Measuring CSF HVA and DOPAC as well as alpha‐synuclein levels may provide an important pharmacodynamic effect of Nilotinib treatment in PD.

The R47H and other variants of triggering receptors on myeloid cells (TREM)‐2, which result in loss of TREM2 function, are strong risk factors for PD.^24^, ^25^, ^26^ Activated microglia in the SN proliferate and produce reactive oxygen species and pro‐inflammatory cytokines, resulting in progressive degeneration of DA neurons in PD.^27^, ^28^ TREM2 may regulate microglial response and phagocytosis. TREM2 inhibits inflammatory responses in microglia via suppression of NF‐kB pathways and activation of innate immunity,^29^ while TREM2 loss of function results in reduced microglial phagocytosis.^30^, ^31^, ^32^ Therefore, measuring TREM2 levels in the CSF may provide another important pharmacodynamic effect indicating neuroinflammation and the phagocytic activity of microglia to potentially reduce alpha‐synuclein levels after treatment of PD patients with Nilotinib.

To determine the pharmacokinetics and pharmacodynamics of Nilotinib in individuals with PD, we designed a physiologically based population pharmacokinetic/pharmacodynamic (popPK/PD) study and measured plasma and CSF concentration of Nilotinib as well as DOPAC, HVA, total and oligomeric alpha‐synuclein and TREM2 levels. Seventy‐five participants were randomized into five groups (n = 15) of an open‐label random single dose (RSD) study that included placebo, 150 mg, 200 mg, 300 mg, and 400 mg Nilotinib. This RSD study in a homogenous cohort of participants with PD provides a valuable insight into the potential mechanisms of action of Nilotinib and its effects on neuroinflammation and potential CSF biomarkers of disease.

## 2 MATERIALS AND METHODS

### 2.1 Study design and objectives

**Figure 1:**
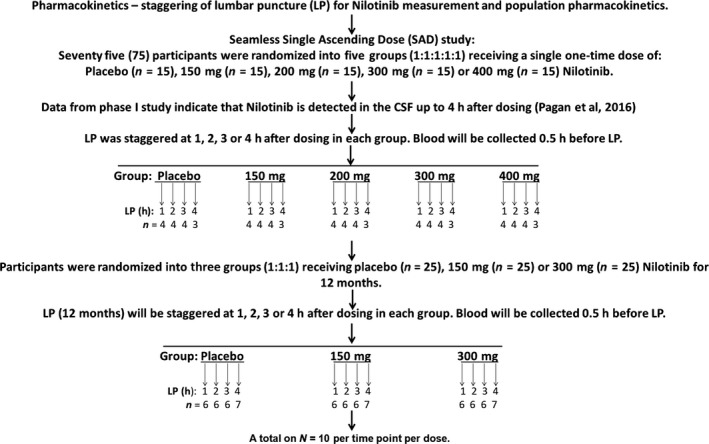
Schematic diagram of a phase II study to determine the pharmacokinetics and pharmacodynamics of Nilotinib in a cohort of 75 participants with Parkinson's disease. The Random Single Dose (RSD) study was performed as part of a randomized double‐blind, placebo controlled study for 1 year. A total of 15 participants were enrolled in each group (placebo:150 mg:200 mg:300 mg:400 mg) in the RSD study and lumbar puncture (LP) was performed in each group in a random fashion at 1, 2, 3 and 4 hours. CSF and plasma were analyzed to determine Nilotinib concentration and biomarkers of disease within each treatment group (dose study) and at each time point (exploratory time‐dependent study)

A total of 100 subjects were screened and 75 individuals were enrolled in a single random dose (RSD) study to determine the pharmacokinetics/pharmacodynamics of Nilotinib. This study was conducted in subjects with PD with Hoehn & Yahr stage between 2.5 and 3. Eligible participants were not receiving any monoamine oxidase (MAO)‐B inhibitors (Rasagiline or Selegiline) for at least 6 weeks prior to dosing and a maximum dose of ≤ 800 mg levodopa (Sinemet/carbidopa/levodopa/entacopone (Stalevo) or IPX066 (Rytary) adjusted to 800 mg carbidopa/levodopa equivalent of 800 mg or less) daily was allowed. Nilotinib has significant drug‐drug interactions with CYP3A inhibitors, inducers, and substrates as well as several other CYPs according to the Novartis Investigator Brochure and patients receiving CYP3A inhibitors were excluded from this study. Montreal Cognitive Assessment (MoCA) score ≥ 22 was used to identify mild cognitive impairment (MCI) at screening. Baseline visits were scheduled 2‐4 weeks after screening and results from all screening procedures were reviewed and all inclusion/exclusion criteria were met prior to baseline assessments. After recruitment, an open‐label RSD study was performed and participants (N = 75) were randomized into five groups (n = 15; 1:1:1:1:1) and received a single daily dose of placebo, 150 mg, 200 mg, 300 mg or 400 mg Nilotinib (Figure 1). Because we previously demonstrated that Nilotinib is detectable up to 4 hours after drug administration in PD and DLB patients,^16^ blood collection and lumbar puncture (LP) were staggered as shown in Figure 1 between 1 and 4 hours and blood was drawn half an hour prior to LP. All LPs were performed roughly within 2 hours from the last levodopa dose. Nilotinib was also taken on an empty stomach at least 2 hours after a meal. Following the 1 week wash‐out period, all participants were randomized into three groups of placebo, 150 mg and 300 mg doses for 12 months (NCT02954978). This double‐blinded, placebo‐controlled study is currently underway and LP and blood will be collected at the end of 1 year treatment to obtain physiological population pharmacokinetics and pharmacodynamics. We performed this seamless, open–label, RSD experiment as part of a larger long‐term study with two active arms (150 mg and 300 mg) and placebo.

### 2.2 Standard protocol approvals, registrations, and patient consents

This study was conducted in accordance with Good Clinical Practice guidelines and was approved by the Institutional Review Board (IRB# 2016‐0380) at Georgetown University Medical Center as well as by the scientific review board at Georgetown‐Howard Universities Center for Clinical and Translational Science (GHUCCTS). Informed consent was obtained from all participants and study partners. The study was conducted under Food and Drug Administration Investigational New Drug (IND) # 123183, and registered in ClinicalTrials.gov (NCT02954978). An external independent Data Safety Review Board (DSMB) that includes a movement disorders neurologist, a biostatistician, a cardiologist, and a clinical pharmacologist, as well as an independent study monitor, were appointed to monitor study safety and progress.

### 2.3 Plasma and CSF collection

We previously showed that Nilotinib was detected in the human CSF 4 hours after administration.^16^ Blood draw (15 mL) and LP (∼15 mL CSF) were performed on all patients roughly 2 hours after they received the last levodopa dose and at 1, 2, 3, or 4  hours after oral administration of Nilotinib. Plasma was isolated immediately after blood draw and aliquoted and stored at −80°C. CSF was aliquoted and stored at −80°C. Freeze/thaw cycles were avoided. To avoid CSF contamination with blood, the first 1  mL of CSF collection was discarded and all samples were centrifuged at 1000 ***g*** for 15 minutes. Samples that contained  >25  ng/mL hemoglobin were eliminated and were not tested for biomarkers. The hemoglobin levels in CSF samples were measured human hemoglobin ELISA Quantitation Set (Cat # E80‐136) Kit (Bethyl Lab Inc, Montgomery, TX) according to the manufacturer's instructions.

### 2.4 Mass spectrometry to evaluate Nilotinib pharmacokinetics

Plasma and CSF samples (20 μL) were thawed initially on ice at room temperature and transferred to Eppendorf tubes containing 100 μL of water. The extraction solvent (500 μL) of acetonitrile/methanol (50:50) containing the internal standard (5 ng/mL of Nilotinib_13C_2H3) was added to the sample. The mixture was vortexed and incubated for 20 min on ice to accelerate protein precipitation and dialysis through 25 μm membranes to obtain unbound or free Nilotinib. After incubation, the samples were vortexed and centrifuged at 13 000 ***g*** for 20 min at 4°C. The supernatant containing unbound Nilotinib was freeze‐dried using speed vacuum and reconstituted in 200 μL of methanol: water (50:50) and processed by mass spectrometry (MS).

The samples were resolved on an Acquity UPLC BEH C18 1.7 μm, 2.1 × 50 mm column online with a triple quadrupole mass spectrometer (Xevo‐TQ‐S, Waters Corporation) operating in the multiple reaction monitoring (MRM) mode. The instrument parameters were optimized to gain maximum specificity and sensitivity of ionization for the parent (m/z = 530.27 Nilotinib) and daughter ions (m/z = 289.01 Nilotinib) using the “IntelliStart” feature of MassLynx software (Waters Corporation). The metabolite ratios were calculated by normalizing the peak area of endogenous metabolites within tissue samples normalized to the internal standard Nilotinib_13C_2H3.

### 2.5 Quantification of DA metabolites DOPAC and HVA by LC‐MS/MS

Concentrations of DOPAC and HVA in the CSF samples were measured by ultrahigh performance liquid chromatography with electrospray tandem mass spectrometry (UHPLC‐MS/MS) following derivatization with benzoyl chloride as previously described.^33^ Briefly, the UHPLC‐MS/MS system included a PAL autosampler (CTC Analytics, Switzerland), an Advance UHPLC pump, and an EVOQ Elite triple quadrupole mass spectrometer (Bruker Daltonics) equipped with an electrospray ionization (EPI) source operating in a positive mode at + 4500 V. The source parameters were as follows: probe gas flow 50, nebulizer gas flow 60, probe temperature + 300 °C, cone gas flow 30, cone temperature + 200 °C, CID gas Ar 1.5 mTorr. The separation of the benzoyl derivatives was achieved on a Luna Omega C18 PS column, 150 × 2.1 mm, 1.6 μm (Phenomenex). Mobile phase A was water, 0.1% (v/v) formic acid, and 6.7 mmol/L ammonium formate. Mobile phase B was acetonitrile and 0.1% formic acid. The gradient elution was as follows (min ‐ %A/%B): 0 – 98/2; 0.2 – 98/2; 6 – 30/70; 7.5 – 30/70; 7.6 – 98/2; 8.2 – 98/2; the flow rate was 400 μL/min. The stable isotope‐labeled DOPAC‐d5 and HVA‐d3 (Toronto Research Chemicals, Canada) were used as the internal standards. The derivatization procedure was performed at room temperature. To a 10 μL volume of CSF sample or a calibration standard in CSF (148 mmol/L NaCl, 4 mmol/L KCl, 0.8 mmol/L MgCl_2_, 1.4 CaCl_2_, 1.2 mmol/L Na_2_HPO_4_, 0.3 mmol/L NaH_2_PO_4_, pH 7.2), the following reagents were pipetted and the mixture was vigorously shaken after each pipetting step: 6 μL of the internal standard mixture (1 μmol/L of each deuterated analyte standard in water), followed by 6 μl of 0.1 mol/L sodium tetraborate buffer and 6 μL benzoyl chloride (1% v/v in acetonitrile, prepared fresh daily). The resulting solution was mixed for 5 min; thereafter, 26 μL of 1% formic acid in acetonitrile was added. The samples were shaken for an additional 3 minutes, centrifuged at 177.0912 *g* for 8 minutes; thereafter, 40 μL of the supernatant was aspirated and the final samples were placed in a vacuum centrifuge miniVac Duo concentrator (Genevac) for 10 minutes in order to reduce the acetonitrile content. Three microliters was injected into the column. The calibration curves were constructed in the range of 0.25‐16 384 nmol/L. The levels of DOPAC and HVA in the CSF samples were measured by Mass Spectroscopy at Pronexus Analytical AB, Bromma, Sweden and was verified by ELISA as detailed below.

### 2.6 Measurement of DA metabolites by ELISA

Human HVA solid phase sandwich ELISA was performed on human CSF samples. A 100 μL CSF or plasma sample is incubated with 100 μL HRP‐conjugate reagent and incubated for 1 hour at 37°C using solid phase sandwich ELISA (MyBioSource, Cat# MBS064661). After washing, 50 μL of chromogen solution A and 50 μL of chromogen solution B are added to the solution and incubated for 15 minutes at 37°C. The reaction is stopped with 50 μL stop solution and the optical density is read at 450 nm. The magnitude of the absorbance is proportional to the quantity of CSF and plasma HVA.

### 2.7 Total alpha‐synuclein ELISA measurement

Solid phase alpha‐synuclein sandwich ELISA (Cat#SIG38974, Biolegend) was performed on CSF and plasma. To avoid freeze‐thaw cycles, immediately after LP and blood draws, 15 ml CSF and 5 ml plasma were aliquoted on ice into 0.5 ml tubes and stored at −80°C. Fresh aliquots were used to perform ELISA. All samples were analyzed side by side using the same reagents. Total alpha‐synuclein rabbit monoclonal antibody (amino acids 118‐123) was coated on the microwells and 200 μL CSF or plasma was added to designated wells. CSF samples were diluted 1:10 while plasma samples were diluted 1:50. After overnight sample incubation at 4°C, alpha‐synuclein was captured by the coated antibody. After washing, a biotinylated mouse monoclonal alpha‐synuclein (amino acids 103‐107) detection antibody was added to each well to detect the captured alpha‐synuclein (amino acids 118‐123). Samples were incubated with 50 μL of detection antibody for 2 hours at room temperature. After washing, 200 μL of streptavidin HRP was added and incubated for 1 hour at room temperature to recognize the bound biotinylated detection antibody. Samples were then washed and incubated with 100 μL of chemiluminescent substrates. Plates were shaken for 10‐15 seconds and read immediately by a luminometer.

### 2.8 Oligomeric alpha‐synuclein ELISA measurement

Solid phase human alpha‐synuclein oligomer sandwich ELISA (Cat# MBS730762, Mybiosource) was performed on CSF. To avoid freeze‐thaw cycles, immediately after LP and blood draws, 15 ml of CSF and 10 mL of plasma were aliquoted on ice into 0.5 ml tubes and stored at −80°C. Fresh aliquots were used to perform ELISA. All samples were analyzed side by side using the same reagents. Fifty microliters of standards or CSF samples was added to the appropriate wells. Five microliters of balance solution was dispensed into samples only and mixed well. One hundred microliters of conjugate was added to each well. The sample solution was mixed well and incubated for 1 hour at 37°C. After washing, samples were incubated with 50 μL substrate A and 50 μL substrate B per well, including blank control well. The sample solution was incubated for 10‐15 minutes at 37°C before 50 μL of stop solution was added to each well, including the blank control well. To determine optical density (O.D.), samples were read at 450 nm using a microplate reader immediately.

### 2.9 Multiplex Xmap TREM2 ELISA

Xmap technology uses magnetic microspheres that are internally coded with two fluorescent dyes. Through precise combinations of these two dyes, multiple proteins are simultaneously measured within a sample. Each of these spheres is coated with a specific capture antibody. The capture antibody binds to the detection antibody and a reporter molecule, completing the reaction on the surface of the bead. All samples were analyzed in parallel using the same reagents. Twenty‐five microliters of human CSF or plasma was incubated overnight at 4°C with 25 μL of a mixed bead solution containing TREM2 (Millipore Cat# HNS2MAG‐95K). After washing, samples were incubated with 25 μL detection antibody solution for 1.5 hours at room temperature. Streptavidin‐phycoerythrin (25 μL) was added to each well containing 25 μL of the detection antibody solution. Samples were then washed and suspended in 100 μL of sheath fluid. Samples were then run on MAGPIX with Xponent software. The median fluorescent intensity (MFI) data were analyzed using a five‐parameter logistic or spline curve‐fitting method for calculating TREM2 concentrations in samples.

### 2.10 Phosphorylated Neurofilament H (pNF‐H) measurement

Solid phase pNF‐H sandwich ELISA (CAT# NS170), Biolegend) was performed on CSF samples collected from the current popPK/PD RSD study and previous phase I open‐label study at baseline and after 6 months of Nilotinib treatment in PD and DLB patients (see study description in reference 16). To avoid freeze‐thaw cycles, immediately after LP and blood draws, 15 ml of CSF was aliquoted on ice into 0.5 ml tubes and stored at −80°C. Fresh aliquots were used to perform ELISA. All samples were analyzed side by side using the same reagents. TBS was added and removed from the wells before adding 50 μL of standards. The plate was incubated for 1 hour at room temperature with gentle shaking. After washing, 100 μL of the diluted anti‐pNF‐H antibody was added to each well. The plate was incubated for 1 hour at room temperature with gentle shaking. After incubation with pNF‐H antibody, samples were washed and 100 μL of the diluted alkaline phosphatase conjugated goat antirabbit polyclonal antibody was added to each well. The plate was incubated for 1 hour at room temperature with gentle shaking before being rinsed with 200 μL of 1× TBS. One hundred microliters of freshly diluted 1× pNPP alkaline phosphatase substrate was then added to each well and incubated in the dark at room temperature for 30‐60 minutes. The plate was immediately read at 405 nm and 50 μL of 3N NaOH was added to each well to stop the reaction.

### 2.11 Experimental design and data analysis

An open‐label RSD study was performed in which participants (N = 75) were randomized into five groups (n = 15; 1:1:1:1:1) and received a single daily dose of placebo, 150 mg, 200 mg, 300 mg, or 400 mg Nilotinib (Figure 1). As Nilotinib is detectable up to 4 hours after drug administration in PD and DLB patients,^16^ blood collection and lumbar puncture (LP) were staggered (Figure 1) between 1 and 4 hours and blood was drawn half an hour prior to LP. Key biomarkers included CSF dopamine metabolites, HVA and DOPAC, alpha‐synuclein, and TREM2, and different doses were compared with placebo. However, further exploratory analysis was performed to compare changes in biomarkers at each time point when CSF was collected to determine whether a single dose of Nilotinib would affect CSF levels of biomarkers in a time‐dependent manner. Data are summarized as mean±SEM (standard error of the mean) for n = 15 in each group when different doses were compared with placebo. All graphs and statistical analyses were performed using Graph Pad Prism Software version 5.01 (Graph Pad Prism Software, Inc., CA). To account for differences between groups, all estimates used for means and SD were based on the mean of relative changes, not the mean value changes. With 15 subjects in each dose group, the normality of biomarkers was examined using the Shapiro‐Wilk test. For the purposes of comparison, we used a one‐tailed Welch *t* test to examine the mean differences between placebo and each treatment with Nilotinib. For pharmacokinetics, the sample allocation for 1, 2, 3, or 4 hours after dosing in each group was randomly done. We also performed an exploratory analysis to better understand the effects of Nilotinib on biomarkers at each time point of CSF collection but due to the small sample size (n = 3‐4), we used a one‐tailed Welch *t* test to boost the power while the statistical significance can be less informative. One sided *P* < 0.05 was considered to be statistically significant. Multiple test correction was not considered due to the relatively small sample size compared with the number of biomarkers. The sample size n and *P*‐values from each test are indicated in each figure. For all HVA and DOPAC experiments, n = 4 for all 1‐hour time points, n = 4 for all 2‐hour time points, and n = 4 for all 3‐hour time points except 200 mg where n = 3. N = 3 for all 4‐hour time points. For total CSF alpha‐synuclein and oligomeric alpha‐synuclein time points, n = 4 (except at 4 hours n = 3), at 1‐hour 200 mg group n = 3, at 2‐hour n = 4 except 400 mg, at 3‐hour n = 4 except 300 mg n = 3. In total, we have 17 time points with n = 3 and 67 time points with n = 4 for our exploratory time‐point analysis.

## 3 RESULTS

### 3.1 Pharmacokinetics of Nilotinib in plasma and CSF

**Figure 2:**
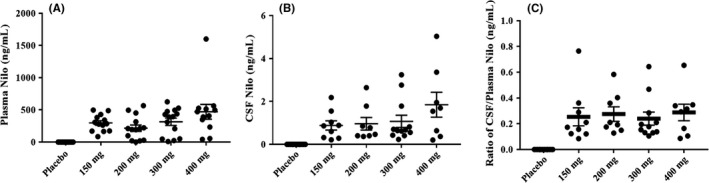
The concentration of Nilotinib in (A) plasma and (B) cerebrospinal fluid (CSF). The ratio of unbound CSF Nilotinib:plasma (C) appears to be the same with all doses and a range of 0.5‐1% Nilotinib is detected in the CSF. N = 15 per group and CSF and plasma were collected 1‐4 hours after drug administration

Nilotinib was not detected in the plasma in the placebo group and the concentration of Nilotinib detected in the plasma (Figure 2A) was similar across all different dose groups of Nilotinib. The 400 mg Nilotinib group had a single patient as an outlier. Similarly, the level of Nilotinib in the CSF (Figure 2B) was similar across all groups compared with the placebo group, with more outliers appearing as the dose increases. These data suggest that Nilotinib enters the brain in a dose‐independent manner. The ratio of unbound CSF: plasma Nilotinib (Figure 2C) appears to be the same in all dose groups and an average of 0.5‐1% Nilotinib is detected in the CSF, suggesting that Nilotinib crosses the BBB and is detected as free or unbound in the CSF at low concentrations.

### 3.2 Effects of Nilotinib on DA metabolism

**Figure 3:**
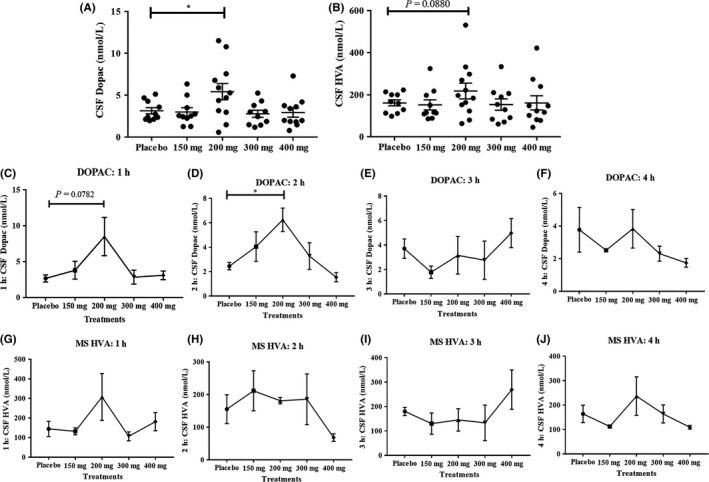
A, The concentration of CSF DOPAC was significantly increased in 200 mg Nilotinib group (n = 15) compared to placebo and all other groups (* indicates *P* < 0.05) and B, CSF HVA trended toward an increase. N = 15 and CSF and plasma were collected 1‐4 hours after drug administration. An exploratory evaluation of CSF DOPAC levels over time show that DOPAC increases at (C) 1 hour and peaks to a significantly high level (D) at 2 hours compared to placebo and all other treatments. DOPAC levels were unchanged at (E) 3 hours and (F) 4 hours after Nilotinib administration. The concentration of HVA was unchanged at all time points (G‐J). The level of HVA and DOPAC in the placebo group was identical. For all HVA and DOPAC experiments n = 4 for all 1‐hour time points, n = 4 for all 2‐hour time points and n = 4 for all 3‐hour time points except 200 mg where n = 3. N = 3 for all 4‐hour time points

To determine the effects of Nilotinib on the metabolism of DA in the brain, the concentration of primary metabolites of DA, including HVA and DOPAC, was measured in the CSF. To normalize the effects of levodopa treatment on the levels of DA metabolism, all data were normalized to 200 mg levodopa, which is the lowest dose that PD participants were receiving as a standard of care. The concentration of CSF DOPAC collected 1‐4 hours after Nilotinib dosing was significantly increased in the 200 mg Nilotinib group (n = 15) compared with placebo and all other groups (Figure 3A). The concentration of CSF HVA collected 1‐4 hours after Nilotinib dosing did not change significantly changed among all treatment groups but trended toward an increase in the 200 mg group (Figure 3B). Further exploratory evaluation of a time‐dependent change in HVA and DOPAC in the CSF of Nilotinib‐treated participants demonstrates that DOPAC increases at 1 hour (Figure 3C) and peaks to a significantly high level at 2 hours (Figure 3D) post Nilotinib administration compared with placebo. DOPAC levels were unchanged at 3 hours (Figure 3E) and 4 hours (Figure 3F) after Nilotinib administration. Furthermore, the concentration of HVA was unchanged at all time points (Figure 3G‐J). However, the CSF level of HVA and DOPAC in the placebo group was identical at all time points, suggesting that exclusion of MOA‐B inhibitors and normalization of levodopa level to 200 mg across all treatment groups provided a control to measure DA metabolites after Nilotinib treatment. For all HVA and DOPAC experiments, n = 4 for all 1‐hour time points, n = 4 for all 2‐hour time points, and n = 4 for all 3‐hour time points except 200 mg where n = 3. N = 3 for all 4‐hour time points.

### 3.3 Effects of Nilotinib on CSF alpha‐synuclein

**Figure 4:**
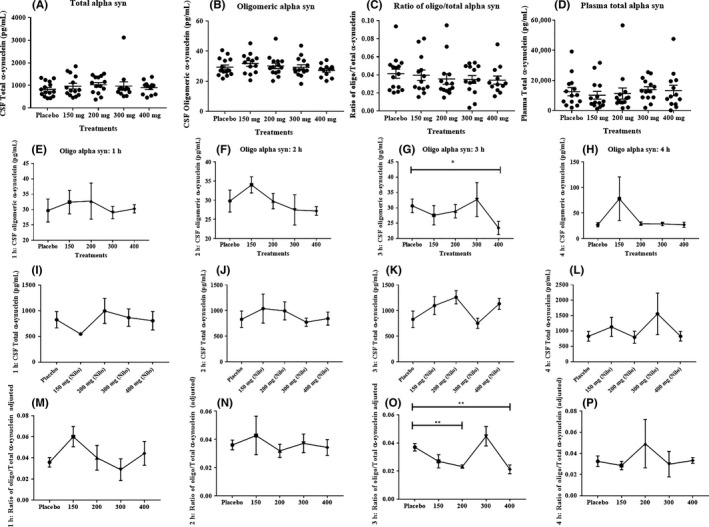
A, The concentration of CSF total alpha‐synuclein was unchanged in all Nilotinib‐treated groups (n = 15) compared to placebo and B, CSF concentration of oligomeric alpha‐synuclein was unchanged in all Nilotinib‐treated groups (n = 15) compared to placebo. C, No difference was observed in oligomeric: total alpha‐synuclein levels in (C) CSF as well as plasma (D). An exploratory evaluation of CSF levels of oligomeric alpha‐synuclein over time shows that oligomeric alpha‐synuclein is not altered at (E) 1 hour (F) 2 hours and (H) 4 hours after Nilotinib administration, but a significant reduction in oligomeric alpha‐synuclein was observed in the 400 mg group after 3 hours (G) of dosing (* indicates *P* < 0.05). No significant changes in total CSF alpha synuclein were observed in all groups and at all time‐points (I‐L). The ratio of oligomeric: total CSF alpha‐synuclein was unaltered at (M) 1 hour and (N) 2 hours and (P) 4 hours after Nilotinib dosing but (O) a significant reduction in oligomeric: total alpha‐synuclein was observed in the 200 mg and 400 mg groups compared to placebo (** indicates *P* < 0.01). For total alpha‐synuclein and oligomeric alpha‐synuclein time points n = 4 (except at 4 hours n = 3), at 1‐hourr 200 mg group n = 3, at 2 hours n = 4 except 400 mg, at 3 hours n = 4 except 300 mg group n = 3

The concentration of total alpha‐synuclein in the CSF was unchanged in all Nilotinib‐treated groups (n = 15) compared with placebo (Figure 4A) and the concentration of oligomeric alpha‐synuclein in the CSF was unchanged in all Nilotinib‐treated groups (n = 15) compared with placebo. No difference was observed in oligomeric: total alpha‐synuclein ratio in CSF (Figure 4C) as well as plasma (Figure 4D). Further exploratory evaluation of CSF levels of oligomeric alpha‐synuclein over time shows that oligomeric alpha‐synuclein is unaltered at 1 hour (Figure 4E), 2 hours (Figure 4F), and 4 hours (Figure 4H) after Nilotinib administration, but a significant reduction in oligomeric alpha‐synuclein was observed in the 400 mg group (Figure 4G) after 3 hours of Nilotinib dosing. For CSF total alpha‐synuclein and oligomeric alpha‐synuclein time points n = 4 (except at 4 hours n = 3), at 1‐hour 200 mg group n = 3, at 2‐hour n = 4 except 400 mg, at 3‐hour n = 4 except 300 mg group n = 3. No changes in total CSF alpha synuclein were observed in all Nilotinib groups and at all time points (Figure 4I‐L) compared with placebo. The ratio of oligomeric: total CSF alpha‐synuclein was unaltered at 1 hour (Figure 4M), 2 hours (Figure 4N), and 4 hours (Figure 4P) after Nilotinib dosing but a significant reduction in oligomeric: total alpha‐synuclein ratio was observed in the 200 mg (Figure 4O) and 400 mg (Figure 4O) groups compared with placebo.

### 3.4 Effects of Nilotinib on plasma alpha‐synuclein

**Figure 5:**
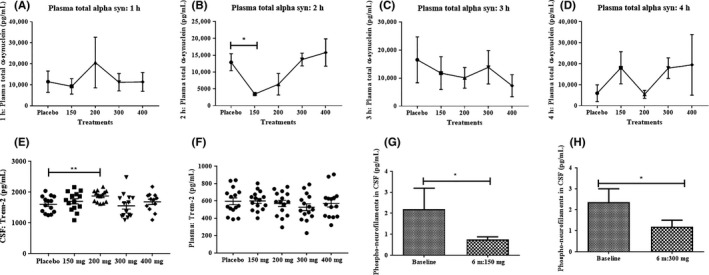
A, The plasma concentration of total alpha‐synuclein was unchanged in all Nilotinib‐treated groups compared to placebo at 1‐hour post Nilotinib dosing. Plasma total alpha‐synuclein (B) was significantly reduced in the 150 mg group (and trended toward a decrease in the 200 mg group) compared to placebo (* indicates *P* < 0.05). C, No difference was observed in total alpha‐synuclein levels at (C) 3 hours (D) and 4 hours compared to placebo. For all plasma alpha‐synuclein time points n = 4 (except at 4 hours n = 3). E, Graph shows TREM2 levels in the CSF (n = 15) and (F) shows TREM2 levels in the plasma (n = 15) after treatment with single dose Nilotinib. G, Graph shows the CSF levels of phosphorylated neurofilaments between baseline and after 6‐months treatment with 150 mg Nilotinib (n = 5) and (H) shows the CSF levels of phosphorylated neurofilaments between baseline and after 6‐months treatment with 300 mg Nilotinib (n = 7)

The plasma concentration of total alpha‐synuclein was unchanged in all Nilotinib‐treated groups (Figure 5A) compared with placebo at 1 hour post Nilotinib dosing. Plasma total alpha‐synuclein was significantly reduced (Figure 5B) in the 150 mg group (and trended toward a decrease in the 200 mg group) compared with placebo. No difference was observed in total alpha‐synuclein levels at 3 hours (Figure 5C) and 4 hours (Figure 5D) compared with placebo. All plasma alpha‐synuclein time points n = 4 (except at 4 hours n = 3). Oligomeric alpha‐synuclein was not detected in the plasma.

### 3.5 Effects of Nilotinib on soluble TREM2 in the CSF

The CSF level of soluble TREM2 shows a slight (10%) increase in the 150 mg Nilotinib group (Figure 5E, n = 15) compared with placebo, and the 200 mg group (n = 15) has significantly increased levels of TREM2 compared with placebo. Both the 300  and 400 mg Nilotinib groups were unchanged compared with placebo (n = 15 per group). The plasma levels of TREM2 were not changed in Nilotinib‐treated groups compared with placebo (Figure 5F), suggesting that Nilotinib at the dose used in this study affects soluble CSF TREM2, independent of its plasma levels.

As expected, after a single dose of Nilotinib, the level of p‐NF‐H was unchanged in all groups in the current study (data not shown). To demonstrate the effects of Nilotinib on markers of cell death, we analyzed p‐HF‐H levels in CSF samples collected from a previous study after treatment with 150 mg (n = 5) and 300 mg (n = 7) Nilotinib for 6 months.^16^ Previous results show that Nilotinib may significantly reduce CSF neuron‐specific enolase (NSE) and S100B, which are markers of neuronal and glial cell death, respectively.^16^ Analysis of CSF samples demonstrates that the level of cell death biomarker p‐NF‐H was significantly reduced between baseline and 6‐month treatment with 150 mg (Figure 5G) and 300 mg (Figure 5H) Nilotinib, suggesting Nilotinib treatment may lead to neuroprotective effects.

## 4 DISCUSSION

The data show that Nilotinib enters the brain and alters surrogate biomarkers of PD in a dose‐independent manner. An equal concentration of unbound free Nilotinib was detected in the CSF of all dose groups, suggesting that perhaps the drug target (c‐Abl, DDRs, etc.) may provide a sink for Nilotinib in the brain. We previously showed that Nilotinib was detected in the human CSF 4 hours after administration but inhibition of CSF Abl extended up to 6 hours,^16^ suggesting that free CSF Nilotinib may not be the final or total concentration of drug that enters the brain. Nilotinib exhibits strong and irreversible binding to its target and the level of CSF Nilotinib may reflect the dynamic pharmacological properties of tyrosine kinase inhibitors via interaction with the ATP‐binding cassette efflux transporters, p‐glycoproteins (PgP), and perhaps aquaporin channels depending on several factors, including individual genetic polymorphisms, gender, ethnicity, and metabolic factors.^34^, ^35^, ^36^ According to the Novartis Investigator Brochure (IB), Nilotinib has a moderate passive permeability and is a PgP inhibitor with an IC50 of 1.7 μmol/L, and this is likely not relevant at the BBB given the low Cmax of 0.06 μmol/L. Furthermore, our mouse studies showed that Nilotinib concentration in the brain increased in an underproportional manner with dose. In humans, plasma concentrations were similar for all doses between 150 and 400 mg. CSF levels and CSF/plasma ratios were also independent of the dose. This is a logical consequence of the constant plasma levels across doses, since the distribution of Nilotinib into the brain depends on plasma levels.

The apparent dose‐independent effect of Nilotinib on potential disease biomarkers in the CSF may be due to the effect of the drug at multiple targets, especially at higher (400 mg) concentrations, suggesting that multi‐target engagement abrogates Nilotinib action and may result in more side‐ and off‐target effects in the CNS. It is possible that a balance between drug level in the CNS and target engagement is best achieved at lower concentrations that lead to more specific binding of Nilotinib to either c‐Abl and/or DDR1/2. We have shown in several experimental models that knockdown of either Abl or DDRs alone results in reduction of alpha‐synuclein, and protection of DA neurons, reminiscent of Nilotinib effects in this study. Nilotinib displays a high binding affinity and potently inhibit Abl (IC50 < 30 nmol/L) and DDRs (IC50 at 3‐6 nmol/L) and it is highly likely that dual inhibition of these receptors facilitates the effects of Nilotinib action. A wider concentration range (50‐600 mg) can be used in order to provide a better insight into both drug concentrations in plasma and CSF and the respective effects on potential biomarkers. Nilotinib plasma levels did not change with dose at least up to 400 mg, suggesting that either patients were underexposed after a single dose or there may be other factors that need to be taken into consideration like solubility‐limited absorption. The observed optimal pharmacodynamics effects at 200 mg suggest that drug concentration may have plateaued at 150‐200 mg. Considering that CSF Nilotinib levels are constant and are likely also due to the constant plasma levels and not to transporter effects at the BBB, plateauing at 150 mg is highly likely and thus efficacy is observed at 200 mg.

We previously demonstrated that Nilotinib treatment for 6 months results in a significant increase in CSF HVA levels in PD and DLB patients from baseline to 2 and 6 months.^16^ The present data show that a single dose of 200 mg Nilotinib results in a significant increase in DOPAC, suggesting that Nilotinib alters DA metabolism. Previous work showed that CSF HVA levels may be unreliable biomarkers for PD (1995).^37^ However, our results show that not only HVA but also DOPAC is altered when participants were off any MOA‐B inhibitors (selegiline and rasagiline) for at least 6 weeks before screening. Additionally, it was recently reported that de NOVO PD patients show a peak of CSF HVA levels around 1.5‐2 hours after administration of 200 mg levodopa and the level of CSF HVA remains constant for a few (up to 4) hours.^38^ In the current study, LPs were performed within 2 hours (on‐state) after last levodopa administration and were staggered for up to 4 hours. The results show that the levels of HVA and DOPAC in the placebo group are unchanged, supporting previous findings^38^ and suggesting a plateau of CNS HVA and DOPAC in the present cohort for at least 4 hours. In the previous study HVA was measured at baseline and 2 months, when MOA‐B inhibitors were eliminated and levodopa treatment was reduced in most patients. However, the level of HVA continued to rise between 2 and 6 months. The single dose effect of Nilotinib on HVA and DOPAC strongly supports alteration of DA metabolism that maybe a long‐term disease modifying effect of Nilotinib in PD.

TREM2 is a receptor expressed on microglia^39^ and plays an important role in neurodegeneration.^40^, ^41^ The R47H and other variants of TREM2, which result in loss of function, are reported to be risk factors for PD.^24^, ^25^, ^26^ The role of TREM2 in neurodegeneration remains controversial^41^, ^42^ as partial or incomplete loss of TREM2 leads to differential effects on microglia and neurodegenerative pathology.^43^ Microglia activation in the SN of the PD brain may produce neurotoxic molecules, resulting in progressive degeneration of DA neurons.^27^, ^28^ TREM2 is implicated in the regulation of a regulatory microglial response.^44^ TREM2 loss of function results in reduced microglial phagocytosis,^30^, ^31^, ^32^ while TREM2 function inhibits inflammatory responses in microglia via suppression of NF‐kB pathways and is strongly implicated in microglia innate immunity.^29^ Overexpression of TREM2 reduces 1‐methyl‐4‐phenyl‐1,2,3,6‐tetrahydropyridine (MPTP)‐induced neuropathology including DA neuron degeneration and neuroinflammation by negatively regulating NF‐κB signaling pathways in mice.^45^ Our results show that a single dose of 200 mg Nilotinib significantly increases soluble CSF TREM2 levels and this increase is concurrent with improved DA metabolism and a potential reduction of oligomeric alpha‐synuclein, in agreement with recent findings.^45^ We previously found that both TREM2 and its adaptor protein DNAX‐activating protein of 12 kDa (DAP12)^46^ are reduced after DDRs knockdown, suggesting that alteration of this tyrosine kinase receptor, which is potently targeted by Nilotinib,^4^, ^5^ may result in alteration of TREM2 signaling. DDRs levels are increased in postmortem PD brains and Nilotinib enhances neurotoxic protein clearance, attenuates cell death and reduces the number of TREM2 + microglia,^47^ suggesting regulation of myeloid‐derived microglia and protection against neurotoxic proteins.

Oligomeric alpha‐synuclein was not detected in the plasma, but we detected a significant effect on total alpha‐synuclein levels with lower single doses (150 and 200 mg) of Nilotinib. Nilotinib significantly reduced the concentration of alpha‐synuclein that is elevated in the blood of PD patients.^48^, ^49^, ^50^, ^51^ These results are consistent with our previous findings that demonstrated Nilotinib effects on lowering blood alpha‐synuclein in mice.^7^ There is evidence that autophagy is disregulated in the blood of PD patients,^52^, ^53^, ^54^, ^55^ and the effects of Nilotinib on autophagy may underlie its effects on plasma alpha‐synuclein. However, a single dose Nilotinib had no effect on total CSF alpha‐synuclein level using the same assay and conditions used in the measurement of plasma alpha‐synuclein. The levels of CSF oligomeric alpha‐synuclein appeared to change particularly with lower concentrations at 3 hours after Nilotinib administration. These data are consistent with increased CSF levels of TREM2, which enhances the phagocytosis function of microglia and suppresses inflammation,^56^ suggesting that reduction in oligomeric alpha‐synuclein may be the result of increased phagocytosis. We previously demonstrated that Nilotinib promotes autophagic clearance of neurotoxic proteins and improves motor and cognitive symptoms in several models of neurodegeneration without evidence of increased inflammation.^6^, ^7^, ^9^, ^11^, ^12^, ^13^, ^57^, ^58^, ^59^ Measurement of CSF alpha‐synuclein with other PD‐related biomarkers like TREM2 and DA metabolism may validate the utility of CSF and/or plasma alpha‐synuclein as a biomarker, not for the diagnosis or progression of PD, but for specific drug effects like Nilotinib. We observed some variability in oligomeric CSF alpha‐synuclein but the combination with TREM2 and DA metabolism as well as plasma alpha‐synuclein suggest that with longer drug exposure oligomeric alpha‐synuclein that longitudinally increases in the CSF of PD patients^17^, ^19^, ^20^, ^21^ may be a reliable measure of Nilotinib effects on changes of PD‐related biomarkers.^60^, ^61^

In conclusion, this study demonstrates that Nilotinib penetrates the BBB and is detected in the CSF in a concentration‐independent manner. An optimal dose of 200 mg Nilotinib potentially induces a concurrent change in DA metabolism, reduction in oligomeric alpha‐synuclein, and elevation of TREM2 levels in the CSF.

## AUTHOR CONTRIBUTIONS

F. P. was the principal investigator. M. L. H processed the bio‐fluid samples. Y. T.‐Y., A. L., E. E. M., N. Y., and N. J. S. performed lumbar puncture and monitored patients. B. W. treated, consented, monitored, and tested patients, S. M. and M. P. performed data management, J. A. and H. H. H. were the outreach coordinators. X. L. and A. J. F. participated in data analysis. S. L. S. oversaw the PK studies. J. A. performed all statistics. C. M. designed the study and wrote the manuscript. All authors read and approved the final manuscript.

## DISCLOSURES

Charbel Moussa is an inventor of several U.S. and International Georgetown University patents to use Nilotinib and other tyrosine kinase inhibitors as a treatment for neurodegenerative diseases. No other authors declare any conflict of interests with this study.
